# Critical Event Debriefing in a Community Hospital

**DOI:** 10.7759/cureus.8822

**Published:** 2020-06-25

**Authors:** Chidiebere V Ugwu, Marsha Medows, Data Don-Pedro, Joseph Chan

**Affiliations:** 1 Pediatrics, Woodhull Medical Center, Brooklyn, USA; 2 Pediatrics, New York University School of Medicine, New York, USA

**Keywords:** debriefing, critical events, resuscitation, feedback

## Abstract

Introduction

Medical error is currently the third major cause of death in the United States after cardiac disease and cancer^. ^A significant number of root cause analyses performed revealed that medical errors are mostly attributed to human errors and communication gaps. Debriefing has been identified as a major tool used in identifying medical errors, improving communication, reviewing team performance, and providing emotional support following a critical event. Despite being aware of the importance of debriefing, most healthcare providers fail to make use of this tool on a regular basis, and very few studies have been conducted in regard to the practice of debriefing. This study ascertains the frequency, current practice, and limitations of debriefing following critical events in a community hospital.

Design/Methods

This was a cross-sectional observational study conducted among attending physicians, physician assistants, residents, and nurses who work in high acuity areas located in the study location. Data on current debriefing practices were obtained and analyzed using descriptive statistics.

Results

A total of 130 respondents participated in this study. Following a critical event in their department, 65 (50%) respondents reported little (<25% of the time) or no practice of debriefing and only 20 (15.4%) respondents reported frequent practice (>75% of the time). Debriefing was done more than once a week as reported by 35 (26.9%) of the respondents and was led by attending physicians 77 (59.2%). The debrief session sometimes occurred immediately following a critical event (46.9%). Although 118 (90%) of the respondents feel that there is a need to receive some training on debriefing, only 51 (39%) of the respondents have received some form of formal training on the practice of debriefing. Among the healthcare providers who had some form of debriefing in their practice, the few debrief sessions held were to discuss medical management, identify problems with systems/processes, and provide emotional support. Increased workload was identified by 92 (70.8%) respondents as the major limitations to the practice of debriefing. Most respondents support that debriefing should be done immediately after a critical event such as death of a patient (123 [94.6%]), trauma resuscitation (108 [83.1%]), cardiopulmonary arrest (122 [93.8%]), and multiple casualty/disasters (95 [73.1%]).

Conclusions

In order to reduce medical errors, hospitals and its management team must create an environment that will encourage all patient care workers to have a debriefing session following every critical event. This can be achieved by organizing formal training, creating a template/format for debriefing, and encouraging all hospital units to make this an integral part of their work process.

## Introduction

The task of managing a critically ill patient can be very demanding for medical personnel working in the intensive care unit and emergency room. One common activity in both settings is resuscitation, which is defined as a series of interventions conducted by a trained team aimed at restoring and/or supporting vital function in a critically ill patient [1]. Due to the complexity of resuscitation processes, patient care is not always delivered optimally. Human systems and occupational sciences literature on the optimization of team performance suggest that debriefing following critical incidents can optimize team performance [2-4].

In the United States, medical error has become the third major cause of death following cardiac disease and cancer [5]. Several root cause analyses performed revealed that medical errors are mostly attributed to errors of commission, omission, and communication [6]. Debriefing offers a healthcare team the opportunity to re-examine the clinical encounter, discuss individual and team performance, identify errors, and develop performance improvement strategies through reflective learning processes [7-9]. Even though real-time clinical event debriefing can be challenging to implement, it has been identified as an important aspect of effective clinical education, quality improvement, and systems learning. Debriefing can also help protect and support those exposed to critical incidents by minimizing abnormal stress responses [10]. Beyond its potential to improve individual and team performance, the International Liaison Committee on Resuscitation (ILCOR) identified the impact of debriefing on actual patient outcomes as an important area of research [11]. Despite these endorsements, this educational intervention is still relatively novel in medicine. Few institutions have formal guidelines and standards on team debriefing after critical incidents such as a failed resuscitation [12-14].

Debriefing is a conversational session that revolves around sharing and examining information after a specific event has taken place. It may follow a simulated or actual experience and provides a forum for the learners to reflect on the experience and learn from their mistakes [15]. Originating from the military and aviation industry, debriefing is used daily to reflect and improve the performance in other high-risk industries. Expert debriefers may facilitate the reflection by asking open-ended questions to probe into the framework of the learners and apply lessons learned to future situations. Debriefing has been proven to improve clinical outcomes such as the return of spontaneous circulation after cardiac arrest and the teaching of teamwork and communication in pediatrics [13].

Debriefing is free of cost and has been perceived by most trainees as useful. It has a benefit of improving behavior and strengthening team cohesiveness for improved quality and safety in everyday clinical practice [15,16].

Even with all these proven benefits, there is a paucity of data on the practice of debriefing among healthcare workers in a community hospital setting.

The aim of this study was to assess the current practice and limitations of debriefing and to ascertain the best timing, effectiveness, need for training, use of established format, and expected goals of debriefing among health care workers in a community hospital.

## Materials and methods

Study design

The researcher in collaboration with other experts designed a 20-question survey that contained inquiries about debriefing after a critical event.

Healthcare workers with direct patient contact were recruited from adult and pediatric emergency rooms, adult intensive care unit, and neonatal intensive care unit, which had a high rate of critical events at the study facility. Staff members working in these areas were approached at random by investigators asking them if they will be interested in participating in a survey on debriefing. The individuals who agreed were taken to a private workspace area (e.g. consulting room behind closed doors) and given more information about the study and a verbal consent obtained. Surveys were administered, giving respondents enough time and privacy to answer questions. This activity was carried out over a period of two months, which was enough to capture most of the healthcare workers in those departments. Participation was voluntary and risk-free (as they were anonymous) and participants were given an option to stop at any time or choose not to answer any of the questions if they felt uncomfortable doing so. This study met the criteria for exempt status after being reviewed by the institutional review board at the study facility.

Outcome measures

Demographic information of each participant (position and years of clinical experience) was obtained. The current practice of debriefing after a critical event was collected, including information on who leads debriefing sessions, how often, how effective, how soon or frequent, and what happens during debriefing sessions.

We also asked participants if they have had any prior training, if there is a need for training, what kind of events should be debriefed, if they had any established format, if they feel debriefing was important, and about their perceived goals and barriers to performing debriefing in their various departments.

Data analysis

The current practice, knowledge, and barriers to debriefing following a critical event in a community hospital were assessed using descriptive statistical analysis. Data were compiled and analyzed using SPSS Statistics Version 25 (IBM Corp., Armonk, NY, USA).

## Results

**Figure 1:**
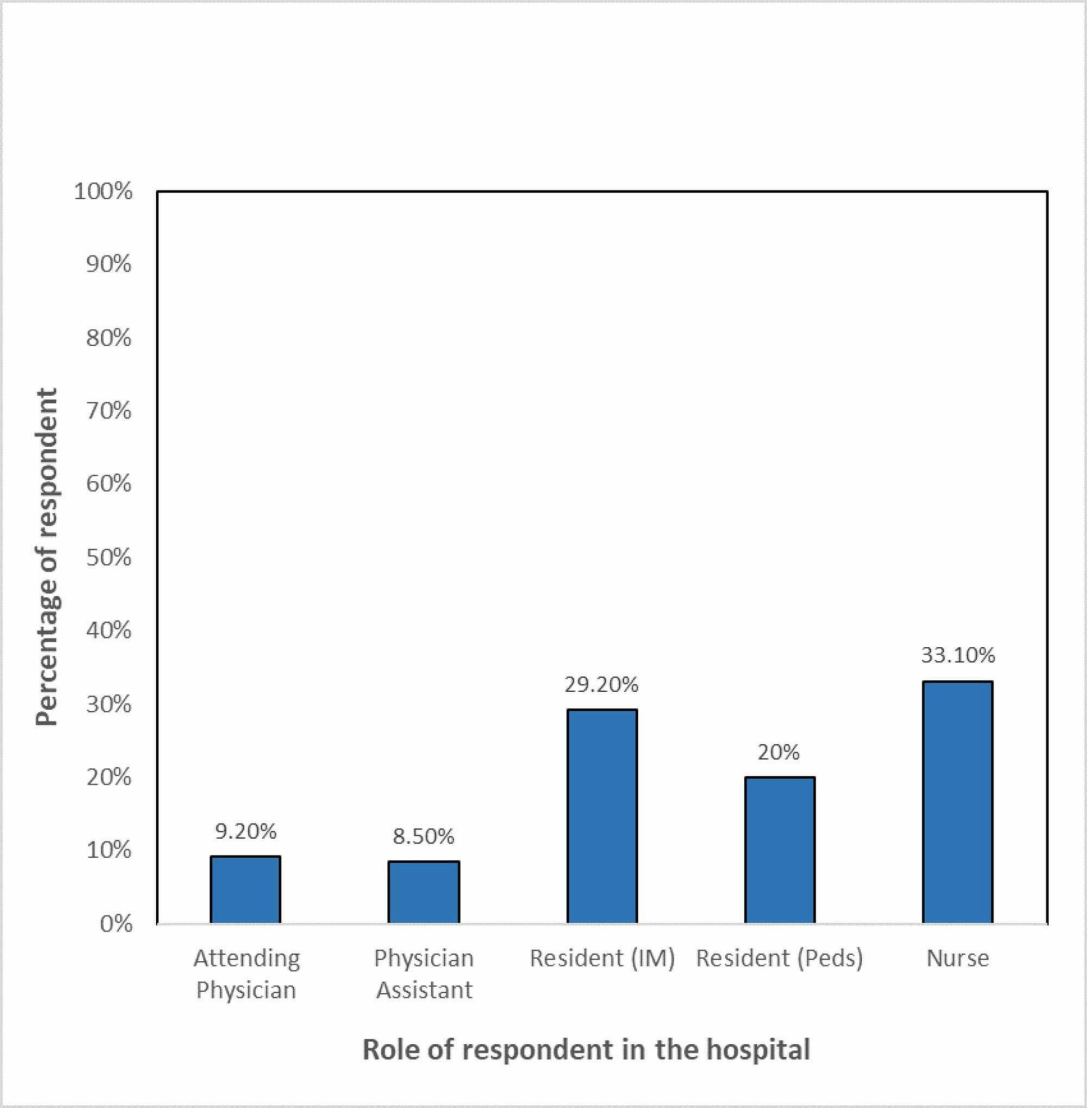
Role of respondents in the hospital.

A total of 130 healthcare workers completed the survey. As presented in Figure 1, majority, i.e., 43 (33%), of all respondents were nurses, whereas 38 (29%) were from internal medicine residents and 26 (20%) were pediatric residents. Most respondents, i.e., 52 (40%), had less than two years of experience, whereas 32 (24%) reported having >10 years’ experience in healthcare.

Practice of debriefing

**Figure 2:**
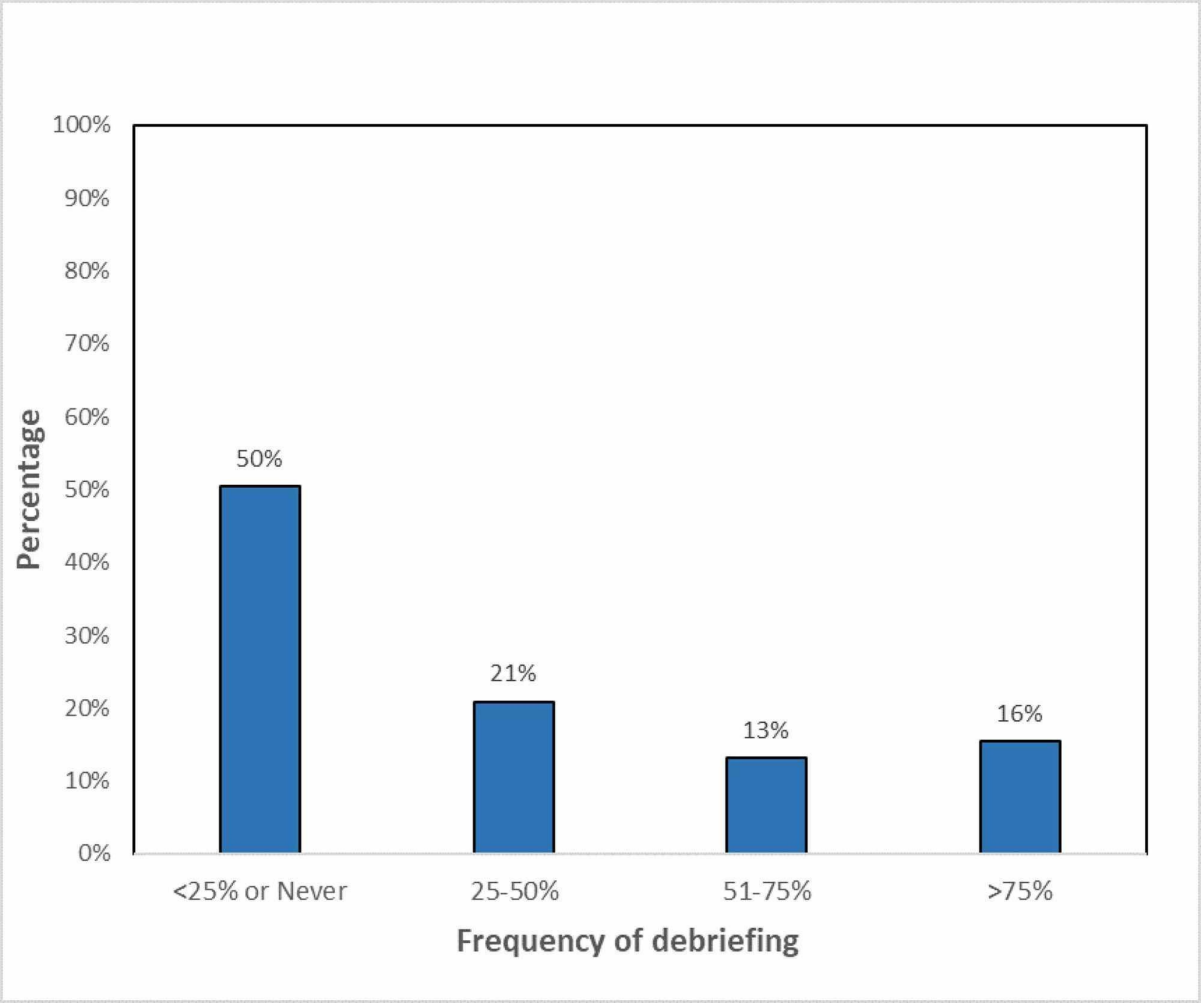
Frequency of debriefing among respondents.

The frequency of debriefing is represented in Figure 2. Most respondents, i.e., 65 (50%), reported to have never/rarely been part of a debriefing session, whereas only 20 (15%) of respondents reported being always engaged in this practice.

The practice of debriefing among healthcare providers in a community hospital is shown in Table 1. A good number of respondents currently debrief immediately after a critical event and are usually led by the attending physician involving mostly clinical members of the team. Discussions were mostly about medical management and identifying problems with systems and processes.

**Table 1:** Practice of debriefing among respondents

Current Practices		n	%
Frequency of critical event in your department?			
	Once a week or more	35	27.3
	Once in two weeks	29	22.7
	Once a month	28	21.9
	Rare (none in a month)	36	28.1
Have you ever received any sort of training on debriefing?			
	Yes	73	61.2
	No	50	38.8
When do debriefings occur?			
	Immediately following the event	61	47.7
	24-72 hours after	30	23.4
	3-7 days	10	7.8
	After a week or later	1	0.8
	Departmental meetings	9	7.0
	Never	17	13.3
How effective are debriefing sessions in your department?			
	Very effective	39	33.9
	Somewhat effective	57	49.6
	Not effective	19	16.5
Who facilitates debriefing in your department?			
	Attending physician	77	64.7
	Residents	23	19.3
	Nurse	7	5.9
	Social worker	1	0.8
	Other hospital staff/anyone	2	1.7
	Nobody	9	7.6
Who attends debriefing sessions in your department?			
	Attending physician		
	Yes	92	70.8
	No	38	29.2
	Physician assistants		
	Yes	57	43.8
	No	73	56.2
	Residents		
	Yes	105	80.8
	No	25	19.2
	Nurses		
	Yes	84	64.6
	No	46	35.4
How effective are debriefing sessions in your department?			
	Always effective	111	85.4
	Somewhat effective	16	12.3
	Barely effective	1	0.8
	I don’t know	2	1.5
Do you think there is a need for training on debriefing at your facility?			
	Yes	118	91.5
	No	11	8.5
Do you have a tool/template/format for debriefing?			
	Yes	12	9.6
	No	113	90.4

Knowledge and attitude towards debriefing

The perception of the ideal practice of debriefing after a critical event is shown in Table 2. Most respondents agree that the practice of debriefing is very useful and is an important tool that will improve patient safety outcome. Majority of respondents also agreed that this practice should be conducted immediately without any delay. Debriefing sessions should be facilitated by an attending physician, and critical events such as death of a patient, cardiopulmonary arrest, multiple casualty/disaster, and trauma resuscitation should be debriefed.​​​​​​​

**Table 2:** Ideal practice of debriefing

Ideal Practice		n	%
When should debriefings be conducted			
	Immediately	102	78.5
	24-72 hours	23	17.7
	3-7 days	3	2.3
	At departmental meetings	2	1.5
Who should facilitate debriefings			
	Attending physician	75	57.7
	Residents	17	13.1
	Nurse	2	1.5
	Social worker	1	0.8
	All healthcare workers	29	22.3
	Trained personnel	5	3.8
What critical events should be debriefed			
	Death of a patient should be debriefed		
	Yes	123	94.6
	No	7	5.4
	Trauma resuscitation should be debriefed		
	Yes	108	83.1
	No	22	16.9
	Cardiopulmonary arrest should be debriefed		
	Yes	122	93.8
	No	8	6.2
	Shock should be debriefed		
	Yes	86	66.2
	No	44	33.8
	Status epilepticus should be debriefed		
	Yes	75	57.7
	No	55	42.3
	Multiple casualty/disasters should be debriefed		
	Yes	95	73.1
	No	35	26.9
	Debriefing is important for patient safety		
	Yes	129	99.2
	No	1	0.8

**Figure 3:**
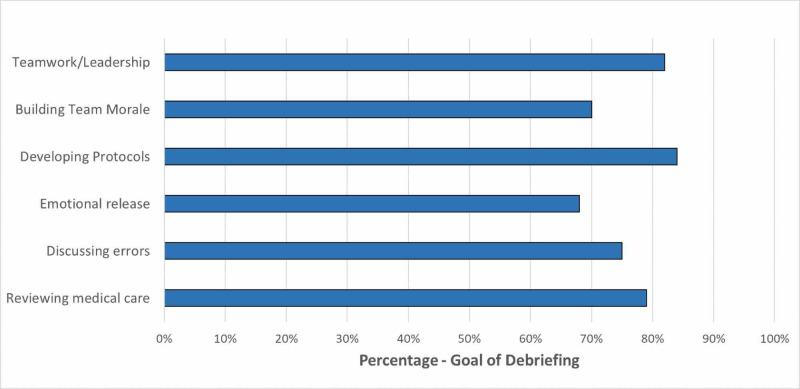
Goal of debriefing.

The respondents reported that the goal of debriefing should be to review medical care, discuss errors, develop guidelines/protocols, discuss teamwork, build team morale, and provide emotional support (Figure 3).

Barriers to debriefing in a community hospital as reported by doctors and nurses are shown in Table 3. Most respondents reported that they have not received any formal training on debriefing and agree that there is a need for one. Majority also reported that there is no template, format, or tool for debriefing. Increased workload was reported by the respondents as the major barrier to debriefing, whereas other barriers include lack of trained facilitators, lack of administrative support, not feeling comfortable because of criticism, and lack of interest by team members.​​​​​​​

**Table 3:** Barriers to debriefing

Barriers	n	%
Workload		
Yes	92	70.8
No	38	29.2
No identified interest/need		
Yes	31	23.8
No	99	76.2
Lack of trained facilitators		
Yes	45	34.6
No	85	65.4
No appropriate setting available		
Yes	27	20.8
No	103	79.2
Not comfortable discussing the event		
Yes	18	13.8
No	112	86.2
Felt criticized/judged		
Yes	30	23.1
No	100	76.9
Too soon or too late		
Yes	27	20.8
No	103	79.2
Lack of administrative support		
Yes	48	36.9
No	82	63.1

## Discussion

Community healthcare workers believe that critical event debriefing provides an avenue to review medical care, discuss errors, develop guidelines, build team morale, and provide emotional support [17-20]. In clinical settings where debriefing is carried out effectively, there is evidence that debriefing sessions can be used as an opportunity to foster learning and help healthcare workers reflect on both their personal and professional values and judgment. Effective debriefing sessions are aided by structure, support, and role-modeling [14].

Although this study revealed that debriefing was done only a few times after a critical event, healthcare workers generally feel that debriefing should always occur after medical trauma or resuscitation [21,22]. Respondents in this study also prioritized multiple casualty/disaster incidents and death of a patient as other events that should be debriefed. These critical events are perceived by healthcare workers as distressing situations, with undesirable emotional impacts, and, in most cases, make the providers a second victim. Most providers agreed that debriefing is an important exercise and that it has the potential to improve patient outcomes.

Similar to other studies, healthcare workers in this community hospital felt that critical event debriefing should happen immediately after the event and should be led by an attending physician [20,23]. The respondents also felt that discussion of medical management and identifying problems with systems and processes were mostly handled during these sessions with less attention to emotional support. This may be due to time constraint and people’s inability/unwillingness to express or communicate their feelings. However, it is important to note that a short review study carried out by Timms in 2019 among emergency department (ED) providers concluded that although there was no evidence about the efficacy of team debriefing in the ED, providers were desirous to have a debrief session after critical events. She also proposed that more research should be carried out to properly ascertain the benefits of debriefing [24].

Findings from this study indicate that most providers have never received any form of training on debriefing and strongly agree to the need for such training. A training like this will equip facilitators on how to run an efficient debriefing session, providing guidance on the key areas that need to be focused on. We also believe that there is a need to inculcate lessons on debriefing into the curriculum of all healthcare workers during their professional training. Additionally, respondents reported that there was no format for debriefing in their departments and that they will prefer to use one. The use of format during debriefing serves as a guide that allows conversations to unfold in an orderly manner, promotes efficient use of time, keeps the discussion on track, and focuses the conversation on important learning objectives [23].

Majority of community healthcare providers reported that barriers to debriefing were in line with those described in previous studies, which were mostly due to increased workload and lack of trained facilitators or established guidelines [20,25]. This finding contrasts with that of a recent study that described communication as the major barrier in their clinical setting [26]. While the busy and ever-changing atmosphere of ED and critical care areas where these events happen remains unpredictable, we believe that providing a structure for timely debriefing in the day-to-day schedule will create an opportunity for less interference with work activity.

Observing the impact and importance of debriefing in reducing medical errors, there is a need for more large studies that are focused on efficacy of debriefing, best formats, setting, and timings, which will yield the highest outcome. Debriefing should be made a core part of medical education for current practitioners and students who are part of the healthcare industry.

Limitations

A cross-sectional study like this is subject to non-response bias; participants had the option to opt out of the study if they did not feel comfortable answering the questions. This can create a bias in the measured outcome. There is a possibility of a recall bias, as respondents were asked to recall their practice of debriefing over an unspecified period. We had a limited number of respondents in this study as it was carried out in a community hospital with a low staff population; therefore, a larger study will be able to portray these practices with increased power. There is also a possibility of a volunteer bias, as the people that agreed to participate in this study may not be representative of the entire population.

## Conclusions

Community healthcare providers rarely practice debriefing even when they know it is an important tool. Although most of them do not have any standardized format or training on debriefing, they believe that debriefing after critical events such as patients’ death and cardiopulmonary resuscitation will improve medical care and patient outcome. Debriefing being a vital tool in healthcare should be made a core part of training curriculum for its professionals.
